# Prognostic effect of peripheral blood cell counts in advanced diffuse large B-cell lymphoma treated with R-CHOP-like chemotherapy: A single institution analysis

**DOI:** 10.3892/ol.2014.2716

**Published:** 2014-11-20

**Authors:** TAKAHIRO YAMAUCHI, TOSHIKI TASAKI, KATSUNORI TAI, SATOSHI IKEGAYA, KAZUTAKA TAKAGI, EIJU NEGORO, SHINJI KISHI, AKIRA YOSHIDA, HIROMICHI IWASAKI, TAKANORI UEDA

**Affiliations:** Department of Hematology and Oncology, Faculty of Medical Sciences, University of Fukui, Yoshida, Fukui 910-1193, Japan

**Keywords:** diffuse large B-cell lymphoma, relapse, R-CHOP, peripheral blood cell counts

## Abstract

The primary objective of the present study was to correlate blood cell counts (lymphocyte, monocyte and platelet counts) with early disease relapse following the attainment of complete remission (CR) by the rituximab, cyclophosphamide, doxorubicin, vincristine and prednisolone (R-CHOP)-like regimen in patients with advanced diffuse large B-cell lymphoma (DLBCL). In total, 30 patients were evaluated, with a median follow-up period of 43 months. All the participating patients attained CR. In total, eight patients experienced relapse within two years of the diagnosis, and the three-year overall survival rate was recorded as 77%. The peripheral counts for lymphocytes, monocytes and platelets, and the lymphocyte-monocyte ratio, all of which have been reported to be prognostic in DLBCL, were assessed. None of these parameters were correlated with the incidence of early relapse or with the prognosis. The lymphocyte count was higher in the patients with durable remission than in those who relapsed, however, no significant differences were identified. Thus, the present study concluded that early disease relapse was not predicted by peripheral blood cell counts in advanced DLBCL that reached CR using the R-CHOP-like regimen.

## Introduction

The most common histological form of non-Hodgkin’s lymphoma is diffuse large B-cell lymphoma (DLBCL), which accounts for ~30% of cases (1). The approved chemotherapy regimen of rituximab, cyclophosphamide, doxorubicin, vincristine and prednisolone (R-CHOP), has established an ~60% three-year overall survival rate in patients, including those with the poorest initial prognosis (2–4). Nevertheless, those patients who experience relapse have a poor disease outlook (5) when high-dose chemotherapy with autologous stem cell transplantation is not provided. Therefore, a detailed prognosis and prediction of the likelihood of relapse may enable the treatment strategy to be optimized for each patient.

The International Prognostic Index (IPI) is the standard tool to determine the prognosis of patients with aggressive non-Hodgkin’s lymphoma (6). The index is deduced from a number of clinical factors, including patient age, performance status, clinical stage, serum lactate dehydrogenase level and the number of extranodal lesions. The original IPI remains useful for stratifying patients with DLBCL who are assigned to rituximab therapy (4,7). The revised IPI (R-IPI) (7) uses identical parameters, but groups them differently in order to identify three risk categories for patients with DLBCL. However, the R-IPI is unable to identify those patients who have a chance of survival of <60% at three years.

Predictors of prognosis for DLBCL are widely sought after (8–10). These include molecular markers identified by gene expression profiling (11), biological markers (12,13) and clinical markers (4,6,7,14–25). Among the clinical markers, and apart from the IPI, blood cell counts and white blood cell differentials have been investigated for their prognostic value in the treatment of DLBCL, since the tests are simple to perform, inexpensive and easily accessible. In previous studies, lymphopenia, a surrogate marker of immune suppression, was found to correlate with patient survival in cases of DLBCL (14–18). Furthermore, other studies identified that the monocyte count, regarded as a surrogate indicator of the tumor microenvironment, was a prognostic factor in DLBCL (19,20). The ratio of lymphocytes to monocytes and the peripheral platelet counts were also reported to be prognostic determinants for DLBCL (21–25).

The present retrospective study was conducted to evaluate peripheral blood cell counts and white blood cell differentials in 30 patients with advanced DLBCL who had achieved complete remission (CR) following 6–8 cycles of standard chemotherapy using R-CHOP-like regimens. The primary objective of the present study was to correlate blood cell counts as predictors with the incidence of early disease relapse. The secondary objective was to identify if these parameters would predict overall patient survival.

## Patients and methods

### Patients

Patients who were admitted to the University of Fukui Hospital (Yoshida, Japan) between 2006 and 2011 were included in the present study. All patients were newly diagnosed with advanced DLBCL (stages III and IV). Diagnosis was based on the pathological findings in biopsy specimens and radiographic determination using computed tomography (CT) and positron emission tomography. Blood cell counts and white blood cell differentials were determined prior to the initiation of chemotherapy. All patients within the study received 6–8 cycles every 21 days of either R-CHOP (375 mg/m^2^ rituximab on day 1, 50 mg/m^2^ doxorubicin on day 1, 750 mg/m^2^ cyclophosphamide on day 1, 1.4 mg/m^2^ vincristine on day 1 and 100 mg prednisolone on days 1–5) or R-tetrahydropyranyl (THP)-COP (375 mg/m^2^ rituximab on day 1, 50 mg/m^2^ THP on day 1, 750 mg/m^2^ cyclophosphamide on day 1, 1.4 mg/m^2^ vincristine on day 1 and 100 mg prednisolone on days 1–5) with a substitution of THP-doxorubicin for doxorubicin. Doxorubicin may produce severe heart toxicity, therefore the THP-COP regime is a preferable choice for elderly patients. Furthermore, the THP-COP regimen exhibits a clinical efficacy that is comparable to the CHOP regimen. The patient’s response to treatment and the incidence of relapse were defined according to the International Workshop criteria for non-Hodgkin’s lymphoma (26). Following the completion of chemotherapy and the confirmation of the achievement of CR, the patients returned periodically for physical examinations, blood tests and CT scans to monitor their disease status. This study was approved by the ethics committee of the University of Fukui (Fukui, Japan).

### Statistical analyses

Overall survival (OS) was calculated from the date of diagnosis until mortality by any cause, or until the date last known to be alive. The OS time was estimated by the Kaplan-Meier method, and the differences were compared using a log-rank test. Graph generation and the statistical analyses were performed using Microsoft Excel 2007 software (Microsoft, Redmond, WA, USA) and GraphPad Prism software (version 6.0; GraphPad Software, Inc., San Diego, CA, USA).

## Results

### Patients

The study population consisted of 30 patients with advanced DLBCL (stages III and IV) who received 6–8 cycles of R-CHOP or R-THP-COP therapy and achieved CR. The patient characteristics, including the clinical stage, IPI, lymphocyte-monocyte ratio and the numbers of lymphocytes, monocytes and platelets, are summarized in Table I. The follow-up period from diagnosis ranged between 11 and 84 months, with a median period of 43 months.

### Blood cell counts and white blood cell differentials according to IPI scores

**Figure 1:**
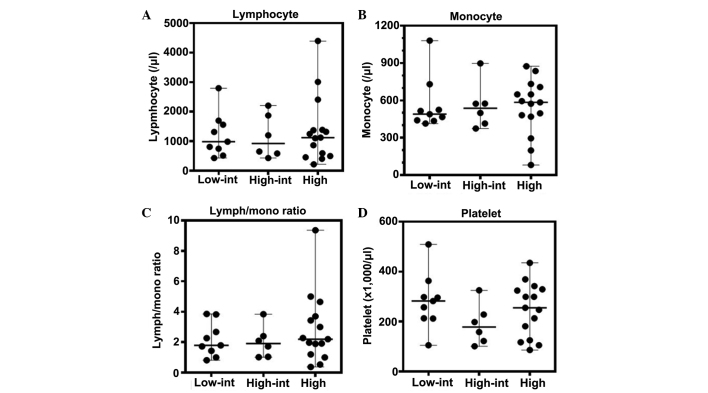
Blood cell counts and International Prognostic Index (IPI) subgroups. Blood cell counts were compared among IPI subgroups. (A) Lymphocyte numbers vs. IPI subgroups, (B) monocyte numbers vs. IPI subgroups, (C) lymphocyte-monocyte ratio vs. IPI subgroups and (D) platelet numbers vs. IPI subgroups. The bars represent the median ± range. Int, intermediate.

**Figure 2:**
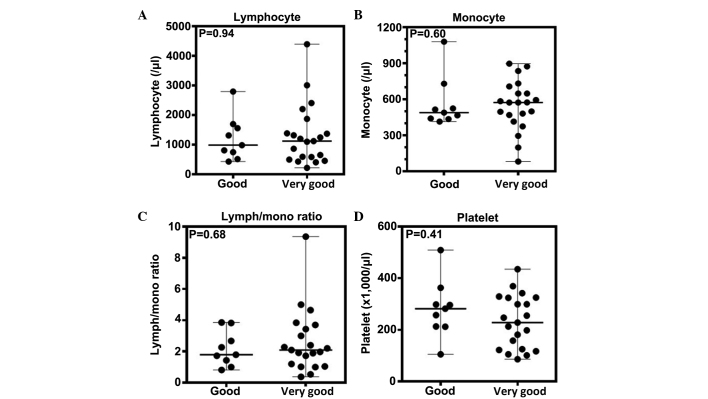
Blood cell counts and revised International Prognostic Index (R-IPI) subgroups. Blood cell counts were compared among R-IPI subgroups. (A) Lymphocyte numbers vs. R-IPI subgroups, (B) monocyte numbers vs. R-IPI subgroups, (C) lymphocyte-monocyte ratio vs. R-IPI subgroups and (D) platelet numbers vs. R-IPI subgroups. The bars represent the median ± range.

The lymphocyte, monocyte and platelet counts, and the lymphocyte-monocyte ratio, all of which have been identified as prognostic markers (14–25), were evaluated for any correlation with the patient IPI scores. No association was identified between each of these values and the IPI scores (Fig. 1). Moreover, these peripheral parameters did not demonstrate any association with the R-IPI scores (Fig. 2).

### Correlation between blood cell counts and early disease relapse

**Figure 3:**
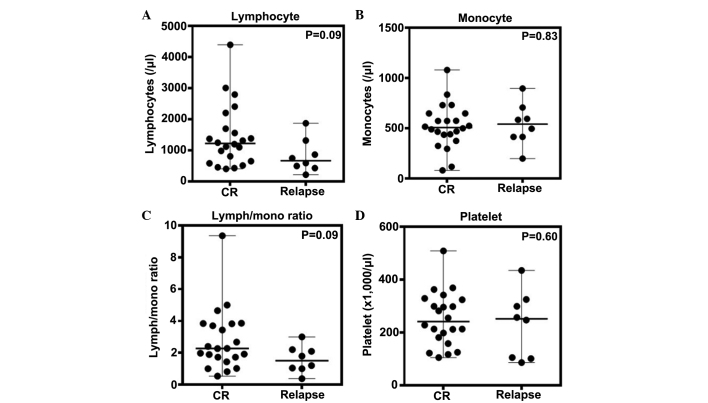
Prediction of early relapse. Among the 30 enrolled patients, relapse within two years of the diagnosis was evaluated for any correlation with blood cell counts at onset. (A) Lymphocyte numbers, (B) monocyte numbers, (C) lymphocyte-monocyte ratio and (D) platelet numbers. The bars represent the median ± range. CR, the patient group with sustained complete remission; relapse, the patient group that experienced relapse.

Of the 30 patients evaluated, eight relapsed within two years of diagnosis, despite achieving CR by the first-line treatment. The blood cell counts were evaluated in terms of a correlation with the occurrence of early relapse; this being relapse within two years from initial diagnosis (Fig. 3). The absolute lymphocyte count appeared to be higher in the patients who maintained CR (median, 1,248/μl) than in the patients who experienced early relapse (median, 747/μl), however, no significant difference was identified (Fig. 3A). In addition, no significant differences were identified between the two patient groups with regard to absolute monocyte count (median, 515/μl for CR group and 540/μl for relapse group), lymphocyte/monocyte ratio (median, 2.3 for CR group and 1.5 for relapse group) and platelet count (median, 239,000/μl for CR group and 251,000/μl for relapse group) (Fig. 3B–D). Thus, these results suggest that the peripheral blood cell counts do not predict the incidence of early disease relapse.

### Survival analysis

**Figure 4:**
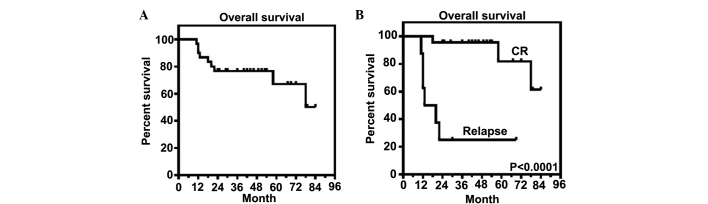
(A) An overall survival curve for all patients. (B) Overall survival curves of the patient group with sustained complete remission (CR) and the patient group that experienced relapse (relapse).

**Figure 5:**
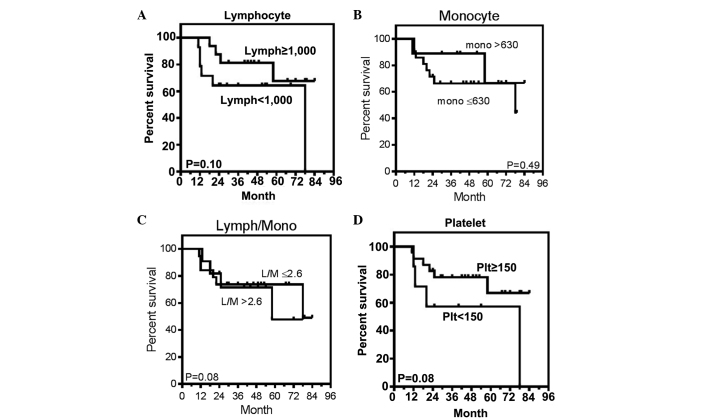
Overall survival curves of the two patient groups divided by the thresholds of (A) lymphocyte number of 1,000/μl, (B) monocyte number of 630/μl, (C) lymphocyte-monocyte ratio of 2.6 and (D) platelet number of 150,000/μl.

According to the Kaplan-Meier method, the three-year overall survival rate for patients with DLBCL was recorded as 77% (Fig. 4A). When the patients were divided into groups for either the attainment of CR or the incidence of early relapse, the CR group had a higher survival rate (Fig. 4B). This suggests that the prediction of early relapse would be critical during initial disease prognosis. The patients were also divided into two groups according to the cut-off values of each of the peripheral counts of lymphocytes, monocytes and platelets, and the lymphocyte-monocyte ratio. The cut-off values were set at 1,000/μl for lymphocytes (14,17), 630/μl for monocytes (20), 2.6 for the lymphocyte/monocyte ratio (22) and 150,000/μl for platelets (25), as determined by previous studies. As shown in Fig. 5, they were not correlated with prognosis. Thus, these results suggested that the peripheral blood cell counts were not predictive of the prognosis of patients with advanced DLBCL who attained CR using standard R-CHOP-like chemotherapy.

## Discussion

The CHOP regimen is the most frequently implemented combination chemotherapy to treat DLBCL, as more intense chemotherapy methods have not demonstrated any additional patient benefits (27). The addition of rituximab to the CHOP regimen has improved the therapeutic outcome, and the superiority of R-CHOP has been confirmed by several clinical studies (2,3). Nevertheless, primary refractoriness is identified in 10–15% of patients, and relapse occurs in 20–25% of those who initially responded to therapy (8,9). In particular, the prediction of relapse may stratify patients according to risk and establish more appropriate treatment schedules, thereby improving clinical outcomes. Numerous prognostic markers have been proposed for patients with DLBCL. These include gene expression profiling, several biological markers, and clinical markers represented by the IPI and its variants (4,6,7,11–13). However, these can be expensive to test and are not always applicable in daily practice. Therefore, inexpensive and readily available parameters are more practical in a clinical context.

The peripheral blood cell count and white blood cell differentials have also been evaluated in terms of their association with the prognosis of DLBCL (14–25). A low absolute lymphocyte count at diagnosis has been revealed to be an important prognostic marker in follicular lymphoma and DLBCL (14–18). Kim *et al* (14) demonstrated a significant impact of an absolute lymphocyte count of <1.0×10^9^/l on event-free survival and OS rates in a group of patients receiving the R-CHOP or CHOP regimen. The two-year OS rates were 80% for the patients with higher lymphocyte counts and 46% for the patients with lower lymphocyte counts (14). Tadmor *et al* (20) assessed the prognostic significance of an absolute monocyte count in 1,017 patients with DLBCL. The best absolute monocyte count cut-off level was <630/mm^3^ and the five-year OS was 71% for these patients, but 59% for those with a count of >630/mm^3^ (P=0.0002) (20). Li *et al* (22) investigated the peripheral blood lymphocyte to monocyte ratio at diagnosis in 438 patients with DLBCL treated with R-CHOP. Using a cut-off value of 2.6, those patients above this threshold demonstrated a better clinical outcome (five-year OS of >80%) than those below it (five-year OS of ~70%) (22). Chen *et al* (25) evaluated the correlation of the peripheral platelet count at disease onset with the therapeutic outcomes of patients with DLBCL treated with the R-CHOP-like regimen. When thrombocytopenia was defined as a platelet count of <150×10^9^/l, the one- and three-year OS rates were 80.2 and 16.7%, respectively, in patients with a platelet count of <150×10^9^/l; these rates were significantly lower than the one- and three-year OS rates of 90.2 and 75.6%, respectively, in those with a platelet count of ≥150×10^9^/l (P=0.0003) (25).

The present retrospective study evaluated peripheral blood cell counts and white blood cell differentials in 30 patients with advanced DLBCL who achieved CR following R-CHOP-like regimens (Table I). The absolute counts of lymphocytes, monocytes and platelets, and the lymphocyte-monocyte ratio were evaluated. However, these parameters were not associated with the IPI or R-IPI scores (Figs. 1 and 2). Furthermore, they were not predictive of early disease relapse and patient OS (Figs. 3–5). The failure to determine any significance of these parameters that have been identified to be of prognostic value in previous studies may be due to the patient selection in the present study. The patients evaluated in this study had advanced DLBCL and had all attained CR by the first-line treatment, while patients at an early disease stage or those with primary refractory disease were excluded. This selection was made due to the importance of predicting the incidence of post-remission relapse in order to stratify treatment schedules to each patient. The small sample size of the present study may also fail to provide significance to a likely parameter, such as the lymphocyte count (Fig. 3).

In conclusion, peripheral blood cell counts were not predictive of disease prognosis or the incidence of early disease relapse of advanced DLBCL that had reached CR using a standard R-CHOP-like regimen. The prediction of relapse following CR would allow for an improved treatment schedule, including the use of up-front high-dose chemotherapy with autologous stem cell transplantation and/or novel investigational agents.

## Figures and Tables

**Table I:** Patient characteristics.

Characteristic	Value
Patients, n	30
Median age at diagnosis, years (range)	72 (56–87)
Gender, n	
Male	19
Female	11
Stage, n	
III	11
IV	19
IPI, n	
Low	0
Low-intermediate	9
High-intermediate	6
High	15
Revised IPI, n	
Very good	0
Good	9
Poor	21
Median no. of peripheral cells	
Lymphocytes, cells/μl (range)	1111 (219–4398)
Monocytes, cells/μl (range)	519 (81–1080)
Platelets, ×10^3^/μl (range)	251 (86–509)

IPI, International Prognostic Index.

## Abbreviations

DLBCL: diffuse large B-cell lymphoma
IPI: International Prognostic Index
CR: complete remission
OS: overall survival
